# Oxidative Stress and Antioxidant Activity in Hypothermia and Rewarming: Can RONS Modulate the Beneficial Effects of Therapeutic Hypothermia?

**DOI:** 10.1155/2013/957054

**Published:** 2013-12-02

**Authors:** Norma Alva, Jesús Palomeque, Teresa Carbonell

**Affiliations:** Departament de Fisiologia i Immunologia, Universitat de Barcelona, Avenida Diagonal 643, 08028 Barcelona, Spain

## Abstract

Hypothermia is a condition in which core temperature drops below the level necessary to maintain bodily functions. The decrease in temperature may disrupt some physiological systems of the body, including alterations in microcirculation and reduction of oxygen supply to tissues. The lack of oxygen can induce the generation of reactive oxygen and nitrogen free radicals (RONS), followed by oxidative stress, and finally, apoptosis and/or necrosis. Furthermore, since the hypothermia is inevitably followed by a rewarming process, we should also consider its effects. Despite hypothermia and rewarming inducing injury, many benefits of hypothermia have been demonstrated when used to preserve brain, cardiac, hepatic, and intestinal function against ischemic injury. This review gives an overview of the effects of hypothermia and rewarming on the oxidant/antioxidant balance and provides hypothesis for the role of reactive oxygen species in therapeutic hypothermia.

## 1. Introduction

Hypothermia has been known as a possible therapeutic tool for millennia. However, it has only been used more systematically in the last two centuries, and it is recently that we have started to understand some of its mechanisms of action and side effects.

### 1.1. Characterization of Hypothermia

Normal body temperature in humans is maintained near a constant level of 36.5–37.5°C through homeostatic processes of thermoregulation. The hypothalamus controls body temperature through the preoptic and the posterior nuclei. The posterior nucleus is especially important, since it acts in regulating the physiological responses that allow the control of body temperature, such as vasoconstriction, shivering or increased intake of food to warm-up, sweating, and vasodilation. Heat is mainly generated in muscle tissue, including other thermogenic organs such as the heart and the liver, while it is lost through the skin (90%) and lungs (10%) and its rate is influenced by the physics involved in the mechanisms of convection, conduction, evaporation, and radiation [1]. In small mammals the brown adipose tissue (BAT) is known to act as a thermogenic organ allowing nonshivering thermogenesis. The presence and physiological relevance of BAT in adult humans were believed to be marginal. In recent years, however, it has been realized that a significant number of adult humans possess active BAT [2, 3] and that cold induces the activation of oxidative metabolism in BAT [4]. Moreover, an inverse relationship between BAT activity and shivering has been demonstrated in humans exposed to cold [5].

When the human body is exposed to cold and the homeostatic mechanisms are unable to compensate the heat that is being lost, there is a drop in body temperature. The symptoms and consequences of hypothermia may vary depending on the degree of hypothermia and have been associated according to the four degrees or stages of severity: mild 32–35°C; moderate, 28–32°C; severe, 20–28°C; and profound at less than 20°C [6]. Other authors such as Marion et al. [7] and Gentinello [8] included an additional category, the extreme hypothermia, when the temperature falls below 14°C.

Symptoms of mild hypothermia may be vague [1] and some physiological responses to preserve heat can be observed with sympathetic nervous system excitation provoking shivering, hypertension, tachycardia, tachypnea, and vasoconstriction. Additional symptoms that may be present are cold diuresis, mental confusion, hepatic dysfunction, and hyperglycemia due to the decrease in glucose uptake by cells, a decrease in insulin secretion, and impaired tissue sensitivity to insulin [6].

Moderate low body temperature results in a stronger shivering. Due to a slower speed in nervous transmission and lower brain blood flow, mild confusion, impaired mental skills, and muscle misscoordination become apparent, and movements are slow and labored [9]. Skin blood vessels contract further as the body focuses its remaining resources on keeping the vital organs warm. Microcirculation alterations cause a reduction of blood flow, red cell sedimentation, and an increase in blood viscosity (2% per degree heat loss), which increases the reduced availability of oxygen in the tissues leading to a hypoxic situation and acidosis [10].

Severe hypothermia occurs with decreasing temperature, and other physiological systems begin to fail: heart rate, breathing rate, and blood pressure decrease all. The hypothalamus is not controlling anymore the thermoregulation. This results in a heart rate of about 30 beats per minute with a temperature of 28°C in humans [6]. Mental skills and motor coordination are still more impaired with a difficulty in speaking, sluggish thinking, incoherent behavior, and amnesia starting to appear; lack of skill in using hands, poor muscle coordination, difficulties in walking, and stumbling are also usually present.

We must also consider the general effects of hypothermia that occur in all categories, such as a decrease in metabolism and oxygen consumption. The basal and activity metabolic rates decrease between 3 and 7% by the fall of 1 degree Celsius [7]. The Q10 or relative change in metabolic rate for every 10 degrees of change in body temperature is about 2.3 [11]. Prakash [12] mentions that oxygen consumption is reduced by 6% for each degree drop in body temperature. Arrhythmias are also often accompanied by frequent atrial fibrillation, bleeding, and coagulopathy due to mismatches in platelet function [8]. Hypothermia has a strong immunosuppressive effect, increasing the risk of infections, specially wound infections and pneumonia [13, 14]. On respiratory function, hypothermia is also inhibitory, initially leading to a rapid shallow breathing followed by a bradypnea, bronchospasm, and hypoventilation. However, the oxygen partial pressure is stable during hypothermia, indicating that both cardiac output and oxygen consumption, despite being reduced, are actually sufficient to meet metabolic needs.

A further problem derives from the subsequent process of rewarming that necessarily follows in some time hypothermia. Rewarming is a challenge for homoeothermic organisms: a rise in temperature implies an increase in metabolism and oxygen demand by tissues. A circulatory collapse characterized by a decreased cardiac output and blood pressure has been described during the rewarming phase in victims of an accidental hypothermia [15]. The final result will be influenced by the rate of rewarming. Polderman and Callaghan [16, 17] advise rates of 0.2–0.5°C/h for cardiac arrest and 0.1-0.2°C/h for other pathologies. After induction of severe hypothermia (20°C) and rewarming in rats a high mortality was found when rewarming at 0.35°C/min, while all animals survived at a rate of 0.25°C/min [18].

### 1.2. The Therapeutic Use of Hypothermia

Despite the undesirable physiological effects of hypothermia, its therapeutic use has been known since ancient times and more recently has been revalued [13, 19, 20].

Mild to moderate hypothermia (35 to 32°C) appears to be useful in preventing tissue damage, cell protection [21], and survival [22]. Several international organizations such as the American Health Association and the International Liaison Committee on Resuscitation have recommended the use of therapeutic hypothermia in patients with cardiac pathologies among others [23]. In the European Resuscitation Council Guidelines [24, 25], induced hypothermia is included in the standard recommendations after cardiopulmonary resuscitation.

Molecular and cellular pathways regulated by hypothermia have been recently reviewed [26]. Many studies on the protective effects of hypothermia have been conducted in cell cultures, for example, in endothelial cells [27], in isolated organs such as heart [28], and in experimental animals [29]. In this regard, the information obtained from studying hibernating mammals is particularly relevant. The hibernating mammals survive cyclical periods of torpor and arousal with large fluctuations in body temperature. It has been suggested that hibernation mitigates apoptosis [30] by an elevation of antiapoptotic [31] or prosurvival [32] proteins. The elucidation of the molecular mechanisms occurring during these periods of hypothermia can be helpful in future human clinical studies of therapeutic hypothermia.

Some of the therapeutic and side effects of the application of hypothermia are well documented [36]. However, it is necessary to clarify the cellular mechanisms induced by cold to enable its safe clinical use. In the present work we discuss the role of RONS and antioxidants during hypothermia and rewarming, and we hypothesize that if the beneficial effects of therapeutic hypothermia could be due to RONS acting as signaling molecules.

## 2. Hypothermia and Oxidative Stress

### 2.1. Tissue Oxygen Availability and Acid/Base Regulation during Hypothermia

**Figure 1:**
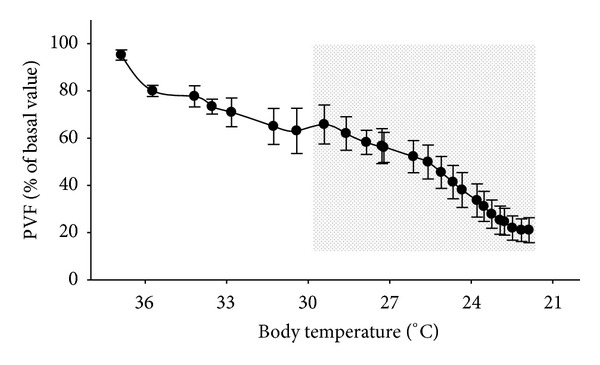
Portal vein flow (PVF) versus body temperature in anesthetized rats during cooling. After induction of anesthesia, animals were placed on a cooling/rewarming table. Animals were cooled at a mean rate of −0.25°C/min. Rats (*n* = 6) were intubated through the trachea and mechanically ventilated with room air. The portal vein circulation was analyzed using a laser-doppler blood flow meter by means of a fiberoptic probe positioned around the portal vein. The curve was calculated by taking the mean of PVF values of all the animals (referred to as a percentage of the starting point) at 5 min intervals and plotting each of these as one point. Note that blood flow is kept close to basal value during the beginning of the cooling (unshaded area) but it dropped drastically under 30 degrees of body temperature (shaded area) [33].

As we mentioned above, the decrease in temperature affects all the physiological systems of the body. The hypothermia process is associated with a reduction of blood flow [37]. It is well known that cold exposure decreases renal [38] and liver flow [39, 40]. We analyzed the correlation between portal vein flows (PVF) as temperature drops from 37°C to 22°C in rats [33].  Figure 1 shows how the cooling caused a decrease in PVF and we can see that the curve of PVF versus temperature fits a second order polynomial regression. This biphasic curve suggests that a homeostatic mechanism is working in opposition to hypothermia and is capable of maintaining PVF in mild and moderate hypothermia close to normal ranges. However, the physiological regulator mechanism can not control body temperature when this decreased towards more severe hypothermic value (below 30°C). Redistribution of blood flow and microcirculation disturbances induced by hypothermia can limit tissue oxygen availability [41].

In addition to changes in the availability of oxygen, hypothermia can affect other blood parameters like partial pressure of gases, electrolytes, and acid/base regulation. Alterations in pH are initially corrected by ventilation and then, in a slower process, by kidneys. When hypothermia develops the pH is altered. In fact, the combination of hypothermia and acidosis is seen as a critical point in the injured by trauma [42]. The failure of respiratory and/or renal functions, acid/base regulation, and ion regulatory mechanisms has been suggested as critical during severe hypothermia [43].

The hemoglobin oxygen affinity actually increases in hypothermia, and a restricted oxygen discharge in tissues could be expected. A reduction in the supply of oxygen to tissues has been reported during mild hypothermia (34°C) [10]. Although lack of oxygen could drive cells to anaerobic metabolism in order to maintain ATP production, cardiac lactate [39], liver lactate [40], and blood lactate [44] kept constant after hypothermia; suggesting that, even though blood supply to tissues is diminished in cold, the oxygen demand also drops in proportion to the lower metabolic requirements.

Accidental or therapeutic hypothermia implies an unavoidable process, the rewarming phase. The rise in temperature will increase metabolism and oxygen demand by tissues. Thus, a mismatch between blood flow restoration and tissue metabolism would cause anaerobic glycolisis, acid overload, and the release of metabolites into the blood stream [40].

As we mentioned above, an important consequence of the hypothermia and rewarming process would be the reduction in oxygen delivery to some tissues. The effect of this decrease in cellular oxygen could resemble a hypoxic condition in which oxygen free radicals are produced [45] and released from the mitochondria [46].

### 2.2. Oxidant/Antioxidant Balance in Hypothermia Rewarming

The reduced availability of oxygen in hypothermia may result in the accumulation of reducing equivalents in the mitochondrial electron transport chain, enhancing the production of reactive oxygen and nitrogen species (RONS) and the resulting oxidative stress, that is, the oxidation and subsequent functional impairment of lipids, proteins, and nucleic acids [47]. Similar to what has been described for hypoxia-reoxygenation process [48], the return to physiological conditions of temperature may lead to an increased production of oxygen free radicals.

It has been reported that hypothermia (24°C to 28°C) can induce an increase in oxidative stress [49–51] but also a decrease in RONS [52, 53]. Using a model of isolated liver preservation, it has been reported that liver perfusion performed at 20°C can enhance the functional integrity of steatotic livers when compared to perfusion at 8°C and 4°C [54]. Perfusion at 20°C also led to a marked improvement in hepatic preservation, cell viability, and reduction in oxidative stress parameters [54, 55]. When considering the effects of hypothermia on oxidative stress, the disparity in the results found could be due to different length of exposition to cold and to the temperature applied.

In physiological conditions there is a balance between the factors that promote the formation of free radicals and the levels of antioxidants. RONS are scavenged by enzymatic antioxidants like superoxide dismutase, glutathione peroxidase, and catalase [56] and by small molecular antioxidants such as reduced glutathione (GSH). GSH appears to be essential for the activation and maintenance of cellular defenses against oxidative stress, since it provides the substrate for glutathione peroxidase to detoxify peroxides. In rats acclimated to cold [57] lipid peroxidation increased and the activities and levels of antioxidants decreased in the erythrocytes.

We have studied the oxidant/antioxidant levels *in vivo* in rats at severe hypothermia and rewarming. After induction of anesthesia, animals were placed on a cooling/rewarming table and were mechanically ventilated with room air. Animals were cooled at a mean rate of −0.25°C/min until they achieve 22°C. After one hour at hypothermia rats were rewarmed at a rate of 0.35°C/min to 37°C. Hypothermia and rewarming increased nitric oxide in plasma and liver and lipid peroxidation in plasma (Table 1). The erythrocyte antioxidant enzymatic activity decreased in hypothermia (superoxide dismutase and catalase) and rewarming (glutathione peroxidase). Results regarding the role of GSH in the hypothermia and rewarming process deserve a more detailed analysis. GSH appears to be essential for the activation and maintenance of cellular defenses against oxidative stress [58] and is the main thiol-disulfide redox buffer in the cell. The data presented in Table 1 indicate that after hypothermia GSH levels are preserved in plasma and also in liver, which is the most important organ for GSH synthesis and exportation into the plasma. In contrast, rewarming results in a high consumption of GSH and, in erythrocytes, a dramatic reduction in the glutathione peroxidase activity. It has been reported that the protective effect of hypothermia against hypoxia-induced damage *in vitro* could be improved with the addition of catalase to the cellular medium [59]. In view of our results, the administration of glutathione seems more advisable, at least *in vivo* situations.

## 3. RONS and Apoptosis

With regard to radical generation there is increasing evidence that cell death is associated with an increase in intracellular RONS [69–72].

Apoptosis, or programmed cell death, is an accurately regulated mechanism whereby the cell actively uses a genetically controlled program to kill itself, with ATP being required to accomplish this process. Apoptosis is a key process that is involved in maintaining tissue homeostasis by removing senescent, genetically damaged cells or cells damaged by disease or noxious agents. Apoptosis is induced by two main ways: the activation of death receptors (the extrinsic pathway) or involving the mitochondria (the intrinsic pathway). In the extrinsic pathway signal arrives when “death ligands” such as TNFa, TRAIL, Apo3L, and Fas ligand (Fas L), bind to their specific membrane receptors causing their intramembrane domains to propagate the death signal intracellularly. RONS have been established as key participants in Fas-induced cell death [70, 71]. In the mitochondrial-mediated pathway the signal is originated intrinsically [73] by the stress produced in organelles like the mitochondria (e.g., RONS) or endoplasmic reticulum (e.g., excess of misfolded proteins), inducing the release of pro-apoptotic factors into the cytosol or inhibiting antiapoptotic molecules that will ultimately trigger apoptosis.

Regardless of the signal origin, the formation of a permeability transition pore at contact sites between the mitochondrial outer and inner membranes and the release of cytochrome c into the cytosol are considered the “points of no return” in apoptotic process. Thus, mitochondria are the central organelle in the execution of apoptosis [74]. RONS generation in mitochondria increased prior to the onset of apoptosis [70], and the apoptotic process could be stopped by the addition of antioxidants [72].

## 4. The Role of RONS as Signaling Molecules

We must bear in mind that the formation of RONS is a physiological process. Indeed, RONS play a critical role in the cell, while at relatively high concentrations they become harmful; low levels can promote cell proliferation and survival [75, 76]. These dual effects of RONS, depending on their concentration, could explain why hypothermia through RONS generation sometimes gets involved in pathologies while when induced previous to or concomitant to an acute damage leads to a cellular protection. Some of the studies regarding the protective effects of hypothermia on oxidant/antioxidant parameters and against different types of injury are summarized in Table 2. While comparing different temperature levels in different species and affecting various tissues, some general conclusions can be drawn. Hypothermia increases oxidative stress, NO levels, and the GSH. When hypothermia is used in a model of injury, like ischemia or hypoxia (known to increase oxidative damage), paradoxically it causes a decreased oxidative stress and the maintenance or improvement of the antioxidant status.

Recently, we have described that the oxidative stress indicators were attenuated in rats with an acute damage (severe hypoxia) at hypothermia (at 22°C) compared with animals at normothermia (at 37°C) [33]. Similarly, in cardiomyocytes [77] it was described a hypothermic protection through a reduction in the hydrogen peroxide-induced damage when cardiomyocytes were incubated at 20°C for 20 minutes. After rewarming, hypothermic cardiomyocytes showed a higher rate of reduction of intracellular reactive oxygen species compared to normothermically maintained cardiomyocytes. In addition, the neuroprotective effects of hypothermia had also been described in a model of oxygen-glucose deprivation in hippocampal cultures at 31°C [78] showing that the protection acts at moderate hypothermia, which may explain the protection observed in the range of therapeutic hypothermia (32–35°C) used in humans.

It has been exhaustively reported that a brief episode of ischemia makes organs (brain, heart, liver, and kidney) remarkably resistant to a subsequent ischemia, phenomenon know as ischemic preconditioning. The mechanisms of signaling pathways in cardiac ischemic preconditioning have been recently reviewed [75] and it is believed that RONS play an important role in the mechanisms of preconditioning and protection. During the early phase of preconditioning ROS and NO production afford protection against further damage in cerebral ischemia [79, 80] and in cardiomyocytes ischemia [81]. The production of adenosine during ischemic preconditioning could mediate the protective effects [82]. Adenosine increases energy production through increased glycolytic flux. Furthermore, it has been observed that adenosine inhibits the release of oxygen radicals during ischemia and reperfusion in myocardial ischemia, thus limiting endothelial cell injury [83]. The production of adenosine during ischemic preconditioning is dependent on the levels of hypoxia-inducible factor 1 (HIF-1) [84] which mediates many adaptive responses to hypoxia by regulating the expression of genes involved in glycolysis, mitochondrial function, cell survival, and resistance to oxidative stress [85]. Interestingly, activation of HIF-1*α* is correlated with better protection of fatty liver grafts after cold storage [86]. However, the addition of an inhibitor of NO in the preservation medium reversed that protection. This highlighted the role of NO in liver preservation.

As referred to hypothermia, our results [33] and others [77–80] lead us to propose the hypothesis that hypothermia through the generation of RONS and increasing GSH can induce protective mechanisms. The most remarkable of hypothermia is that protection is observed both when induced prior to injury, as a preconditioning model (experimental hypothermia), and when applied after damage (therapeutic hypothermia). In the latter case, the sooner hypothermia is applied, the better prognosis patient will have [17, 87].

## 5. Role of RONS in the Beneficial Effects of Therapeutic Hypothermia

Therapeutic hypothermia has been used in the critically ill patients, and there is also abundant evidence from animal models of the protection induced by hypothermia when applied within minutes following ischemic damage (see Table 2). So far, the protective effects of hypothermia are believed to be a consequence of a reduction in the cellular metabolism and the retardation of destructive enzymatic reactions and the concomitant oxygen needs, thus conserving ATP levels [88].

**Figure 2:**
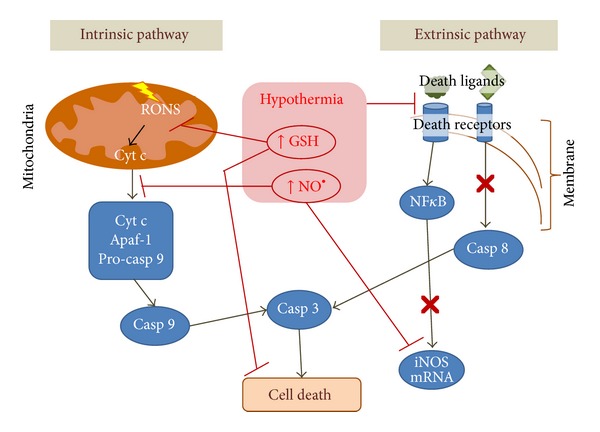
Modulation of apoptosis by hypothermia. After a serious insult the cell can trigger apoptosis, a highly regulated cell death mechanism. *Intrinsic Pathway*. Hypothermia increases ATP stores and slows ion channels then maintaining the integrity of the membranes. Hypothermia applied together or immediately after injury decreases the production of ROS. These events limit the rupture of the outer mitochondrial membrane and the release of proapoptotic molecules like cytochrome c into the cytosol. The hypothermia-induced increase in nitric oxide also avoids cytochrome c release and it is even reported that early NO production can exert a negative feedback regulation of iNOS [34]. Moreover, iNOS transcription activated by NF*κ*B was diminished after hypothermia [35]. Since catalase is absent in mitochondria, maintaining GSH redox cycle is critical to avoid H_2_O_2_ accumulation. There is abundant evidence that hypothermia keeps GSH pool. *Extrinsic Pathway*. It was found that hypothermia decreases the affinity of the death ligands-death receptors, with the consequent inhibition of the initiator caspases like caspase-8 or the NF*κ*B-family molecules.

More recently, the beneficial effects of hypothermia, when applied to prevent an ischemic episode, included a trigger level of RONS that can act as a mechanism for induction of signaling pathways and the modulation of the extrinsic and intrinsic pathways of apoptosis (see Figure 2). Hypothermia does not simply block cell signaling pathway of apoptosis and necrosis but selectively upregulates some protective genes after ischemia [89]. Many experimental assays showed that when hypothermia is applied during an ischemia or hypoxia episode, it is able to inhibit proapoptotic molecules and to induce an increase in antiapoptotic ones in ischemic tissues [90, 91].

Because of these different mechanisms of action, it can be suggested that hypothermia may be protective in many organs and against many kinds of injury.

## 6. Conclusion

The generation of RONS is a typical feature of hypothermia and more prominent in rewarming. There is increasing evidence showing that the beneficial effects of hypothermia included a trigger level of RONS that can act as a mechanism for induction of signaling pathways and the modulation of apoptosis.

## Figures and Tables

**Table 1:** Oxidant/antioxidant status in rats after severe hypothermia and rewarming.

	Arterial Blood			Liver		
	Sham	Hypothermia	Rewarming	Sham	Hypothermia	Rewarming
NOx	10.76 ± 0.52	14.68 ± 0.59***	12.57 ± 0.48^∗,+^	1.28 ± 0.06	1.56 ± 0.06*	2.00 ± 0.10^∗∗∗,++^
TBARS	3.60 ± 0.10	4.66 ± 0.36*	4.46 ± 0.20*	3.93 ± 0.36	4.32 ± 0.43	3.30 ± 0.31
GSH	221.02 ± 5.53	230.61 ± 8.24	140.42 ± 7.50^∗∗∗,+++^	4.56 ± 0.37	4.50 ± 0.17	2.91 ± 0.17^∗∗∗,+++^
SOD	100 ± 3.58	82.86 ± 1.95**	85.81 ± 4.35*	100 ± 3.11	102.80 ± 3.59	100.80 ± 5.73
GPx	100 ± 3.09	101.94 ± 3.38	57.41 ± 7.28^∗∗∗,+++^	100 ± 2.91	99.54 ± 6.87	92.27 ± 2.16
CAT	100 ± 7.54	71.45 ± 4.39**	61.18 ± 4.73***	100 ± 5.34	90.50 ± 6.22	90.34 ± 2.90

Animals were assigned to 3 groups of 6 individuals each. Sham animals were killed after anesthesia. In hypothermia group anesthetized animals were cooled for one hour at a mean rate of −0.25°C /min to achieve 22°C. Then they were killed. The rewarming group was cooled as described above and then it was rewarmed at a rate of 0.35°C/min to 37°C. Oxidative indicators were the concentration of nitric oxide derivatives (NOx) in plasma (nM) and liver (nmol/mg protein) and thiobarbituric acid-reactive substances (TBARS) in plasma (nM) and liver (nmol/mg protein). Antioxidant status was evaluated as thiols in plasma (GSH, *μ*M) and in liver (GSH, *μ*mol/g liver). The enzymatic antioxidant activities of Cu-Zn superoxide dismutase (SOD), glutathione peroxidase (GPx), and catalase (CAT) were evaluated in erythrocytes and in liver and were expressed as a percentage of corresponding sham value. Data is mean ± SEM of six animals. Significantly different from corresponding sham values: *P < 0.05, **P < 0.01, and ***P < 0.001. Significantly different from corresponding hypothermia values: ^+^ *P* < 0.05, ^++^ *P* < 0.01, and ^+++^ *P* < 0.001.

**Table 2:** Selected data showing the protection induced by the experimental hypothermia. This table summarizes information about the impact of the experimental hypothermia on oxidant/antioxidant parameters. Despite the different levels of hypothermia, animal species, tissues, or injury models, the overall effects are as follows. First hypothermia by itself induces an increase in oxidative stress markers and in the reduced glutathione. Second, if hypothermia is applied during another injury there is a decrease in oxidative stress and the maintenance or improvement of antioxidants.

HT level (°C)	Specie	Target	Injury model	Oxidative stress indicator	Injury effects	HT alone effects	HT-induced protection	Comments	Reference
17	Guinea pig	Heart *In vitro *	I/R	ROS generation NADH^+^ Mitochondrial Ca^++^	↑ ↑ ↑	↑ ↑ ↑	↓ ↓ ↓		[28]

20–22	Rat	*In vivo *	Hx (10% O_2_)	Plasma TBARS Liver TBARS Liver GSH/GSSG	↑ ↑ ↓	↑ = ↑	↓ ↓ ↑	Intrahypoxic HT Ventilatory support	[33]

23-24	Rat	*In vivo *	—	Catalase Vitamin E	ND ND	↓ ↓	ND ND	Ventilatory support not provided	[46]

25	Pig	Artery *In vitro *	—	NO synthesis	ND	↑	ND		[60]

26	Rat	*In vivo *	I/R	TBARS SOD Catalase	= = ↑	= ↑ =	↓ = ↓	Intraischemic HT followed by reperfusion at 37°C	[61]

30–32	Rat	Intestine *In vivo *	I/R	TBARS GSH/GSSG Plasma NOx	↑ ↓ ↑	= = =	↓ ↓ ↓	Ventilatory support using a mixture of O_2_/NO^*∙*^	[62]

32	Rat	*In vivo *	Heatstroke (40°C)	O_2_^∙−^ generation MDA in liver	↑ ↑	ND ND	↓ ↓		[63]
	Mouse	CA1 hippocampus	MCAO	NOS expression CaM-KII	= ↓	ND ND	= ↑	HT increases CaM-KII	[64]

33–35	Pig	Brain	MCAO	GSH N-Acetyl aspartate	↓ ↓	ND ND	↑ ↑	Decreasing NAA = cell dysfunction and neuronal loss	[65]
	Rat	*In vivo *	MCAO	iNOS expression Nitrotyrosine	↑ ↑	ND ND	↓ ↓	Intraischemic HT is more effective than postischemic HT	[35, 66]

34	Rat	Isolated Liver *In vitro *	Ischemia	Efflux rate TBARS	↑	=	↓		[67]

35	Mouse	Brain *In vivo *	ALF	GSH/GSSG	↓	ND	↑	GSH/GSSG Incremented over sham values	[68]

ALF: acute liver failure.

CaM-KII: Ca^2+^/calmodulin protein kinase II.

GSH/GSSG: reduced/oxidized glutathione.

HT: hypothermia.

Hx: hypoxia.

I/R: ischemia/reperfusion.

MCAO: middle cerebral artery occlusion.

MDA: malondialdehyde.

ND: not described.

NOS: nitric oxide synthase.

NOx: nitric oxide derived products.

O_2_^∙−^: superoxide radical.

SOD: superoxide dismutase.

TBARS: thiobarbituric acid-reactive substances.

↑, ↓: increase, decrease, respectively.
